# The effects of balanced crystalloid *versus* plasma on endothelial injury, systemic inflammation, and coagulation in experimental endotoxaemia: a randomised human volunteer study

**DOI:** 10.1016/j.bja.2025.08.060

**Published:** 2025-10-18

**Authors:** Daan P. van den Brink, Bashar N. Hilderink, Derek J.B. Kleinveld, Robert B. Klanderman, Inge Pareyn, Karen Vanhoorelbeke, Philip C. Spinella, Nicole P. Juffermans

**Affiliations:** 1 Department of Intensive Care Medicine, Amsterdam UMC, University of Amsterdam, Amsterdam, The Netherlands; 2 Laboratory of Experimental Intensive Care and Anaesthesiology, Amsterdam UMC, University of Amsterdam, Amsterdam, The Netherlands; 3 Department of Intensive Care Medicine, Erasmus MC, Erasmus University of Rotterdam, Rotterdam, The Netherlands; 4 Department of Anaesthesiology, Erasmus MC, Erasmus University Rotterdam, Rotterdam, The Netherlands; 5 Department of Anaesthesiology, Amsterdam UMC, University of Amsterdam, Amsterdam, The Netherlands; 6 Laboratory for Thrombosis Research, KU Leuven Campus Kulak Kortrijk, Kortrijk, Belgium; 7 Trauma and Transfusion Medicine Research Centre, Department of Surgery, University of Pittsburgh, Pittsburgh, PA, USA

**Keywords:** endothelium, endotoxaemia, fluid resuscitation, glycocalyx, inflammation, plasma

## Abstract

**Background:**

During shock, neutrophil-mediated glycocalyx degradation can lead to exposure of the procoagulant endothelial surface and consequent organ injury. Resuscitation using crystalloid fluids may augment this endothelial damage, while resuscitation using plasma may preserve glycocalyx health. We hypothesised that resuscitation with plasma would preserve glycocalyx integrity by reducing inflammation, glycocalyx shedding, and dyscoagulation in a controlled model of systemic inflammation in human volunteers.

**Methods:**

Twelve healthy male volunteers were injected with 2 ng kg^−1^ lipopolysaccharide (LPS) to induce systemic inflammation. Thirty minutes after LPS administration, participants were randomised to receive 10 ml kg^−1^ of either balanced salt solution or solvent detergent plasma (SDP). Plasma syndecan-1, a marker of glycocalyx degradation, was the primary outcome. Additionally, plasma biomarkers of inflammation, neutrophil extracellular trap formation, glycocalyx degradation, endothelial injury, and coagulation were measured serially, by bead-based or enzyme-linked immunoassays.

**Results:**

LPS induced a systemic inflammatory response, accompanied by endothelial injury as indicated by increased levels of syndecan-1 (median increase: 2920–4430 pg ml^−1^, *P*<0.05). Participants receiving SDP had lower levels of neutrophils and plasma matrix metalloproteinase-9, compared with those receiving balanced salt solution. Neutrophil extracellular trap formation, inflammation, and glycocalyx degradation were not affected by fluid therapy. SDP reduced LPS-induced prolonged prothrombin time, but had no impact on other biomarkers of coagulation.

**Conclusions:**

In human endotoxaemia, plasma resuscitation reduced leucocyte and neutrophil levels but did not reduce glycocalyx degradation, when compared with equal volume resuscitation with a balanced crystalloid solution.

**Editor’s key points**

• Organ injury is promoted by neutrophil-mediated glycocalyx degradation, which exposes the procoagulant endothelial surface.
• Laboratory models suggest that plasma may avert endothelial damage observed using crystalloid fluid resuscitation.
• In a cutting-edge human volunteer model, low-dose lipopolysaccharide was infused to generate systemic inflammation and endothelial injury, before participants received either balanced salt solution or solvent detergent plasma.
• Plasma resuscitation reduced systemic inflammation after endotoxaemia, but did not affect glycocalyx degradation.

In critically ill patients, dysregulated inflammation can impair endothelial barrier function. In response to triggers such as an infection or major surgery, activated neutrophils release neutrophil extracellular traps (NETs), including enzymes such as myeloperoxidase (MPO) and neutrophil elastase (ELA2), in a process termed NETosis.^1^ These enzymes interact via matrix metalloproteinases (MMPs) with the endothelium and endothelial glycocalyx, a gel-like layer of carbohydrates covering the luminal side of the endothelial barrier. As a result the endothelial glycocalyx degrades, releasing breakdown products such as syndecan-1 and heparan sulphate into the circulation.^2^ Degradation of the glycocalyx can lead to exposure of the endothelial pro-inflammatory and procoagulant surface, with shedding of excessive amounts of von Willebrand factor (vWF) and formation of immunothrombi.^3^^,^^4^ These changes lead to disturbed microcirculatory perfusion and reduced tissue oxygenation, ultimately resulting in organ dysfunction.^5^ Thus, therapies targeting endothelial barrier dysfunction may improve outcomes in critically ill patients.

During the initial phase of critical illness, fluid resuscitation is often required to ensure adequate tissue perfusion. Clinical guidelines recommend balanced crystalloids as the standard resuscitation fluid of choice.^6^^,^^7^ However, low-protein content fluids, including crystalloids, may be harmful to the endothelium as they induce glycocalyx degradation and endothelial dysfunction *in vitro*.^8^^,^^9^ In experimental shock models, resuscitation with albumin or plasma ameliorates endothelial dysfunction, compared with crystalloids.^10^^,^^11^ In contrast, in clinical trials in critically ill and patients with sepsis, albumin did not reduce organ failure or mortality.^12^^,^^13^ However, in patients with pre-existing hypoabluminaemia, or those who require large resuscitation volumes, a beneficial effect of albumin cannot be ruled out.^14^ It has been hypothesised that plasma may be superior to albumin for preserving glycocalyx function,^15^^,^^16^ which may be attributable to its' protein content or through supplementation of specific factors, such as thrombospondin type 1 motif, member 13 (ADAMTS-13).^17^^,^^18^ In animal models of sepsis and haemorrhagic shock, plasma transfusion restores glycocalyx thickness and reduces endothelial permeability, compared with both crystalloids and albumin.^10^^,^^19^, ^20^, ^21^, ^22^, ^23^, ^24^ In critically ill and injured patients, plasma transfusion has been linked to decreased levels of syndecan-1 and pro-inflammatory cytokines.^25^^,^^26^

Experimental human endotoxaemia induced by lipopolysaccharide (LPS) causes an systemic inflammatory response with glycocalyx degradation. In this study, we investigated the effects of an equal volume of plasma *vs* crystalloid infusion on the development of endotheliopathy, inflammation, and coagulation in a human volunteer LPS-induced endotoxaemia model. We hypothesised that fluid therapy with plasma is superior to crystalloids, in terms of inflammatory response with preservation of the glycocalyx.

## Methods

### Study design

This study was reviewed and approved on April 5, 2022, by the Medical Ethical Committee Amsterdam University Medical Centre (NL74983.018.21), in accordance with the Declaration of Helsinki and Good Clinical Practice. The ‘Resuscitation of Endothelial Permeability in Endotoxemia’ study was a human volunteer, open-label, randomised study conducted at the Amsterdam University Medical Centre. All enrolled volunteers provided written informed consent before enrolment. The study was conducted and reported in accordance with the CONSORT guidelines.^27^

### Inclusion criteria

Healthy male volunteers, aged 18–35 yr, with a body mass index between 20 and 25 kg m^−2^ were eligible (Supplementary Table 1).

### Exclusion criteria

Exclusion criteria were lack of informed consent, abnormal laboratory or urine test screening results, any prescription medications, blood donation or participation in another medical study within the preceding 3 months, previous blood transfusion, previous experimental exposure to LPS, and fever at screening or immediately before LPS infusion (Supplementary Table 1).

### Blinding and randomisation

A randomisation sequence was generated using randomizer.org ^28^ and placed into consecutively numbered opaque envelopes, which were opened on the morning of the experiment. Researchers were not blinded, as participants receiving solvent detergent plasma (SDP) needed extra blood tests for blood product compatibility. Also, it was not feasible to blind for balanced salt solution (BSS) or SDP administration. The plasma product was Omniplasma (Octapharma GmbH, Lachen, Switzerland), which is pooled from 600–1200 donors, before solvent detergent treatment and prion-reduced, and used as a standard plasma product nationwide in the Netherlands. Plasma products were freshly thawed and sourced from the local blood bank of the Amsterdam UMC on the day of the experiments.

### Human volunteer endotoxaemia model

Volunteers were admitted to the research unit of the intensive care department of the Amsterdam UMC and were continuously monitored during the entirety of the experiment. Each subject received a radial artery catheter, for measurement of blood pressure and blood sampling. A peripheral intravenous catheter was inserted for the administration of study products. Vital parameters were continuously monitored using five-lead ECG and pulse oximetry (Philips, Eindhoven, The Netherlands). Temperature was measured hourly using a tympanic thermometer (Genius II, Medtronic, Minneapolis, MN, USA). After measuring all baseline values, 2 ng kg^−1^ LPS from *Escherichia coli O113* (National Institute of Health Clinical Centre, Bethesda, MD, USA) was injected. Thirty minutes after LPS injection, the intervention was commenced, infusion products were adminstered over 1 hour. The experiment concluded 8.5 h after the LPS infusion, by which time systemic symptoms have typically resolved.^29^

### Intervention

Participants were allocated to receive either 10 ml kg^−1^ infusion of BSS (Plasma-Lyte 148, Baxter, Deerfield, IL, USA) or 10 ml kg^−1^ allogeneic SDP (Omniplasma). For safety reasons, the initial two participants received BSS and the subsequent two received SDP. The remaining participants were randomly distributed between both groups.

### Sample collection

**Fig 1:**
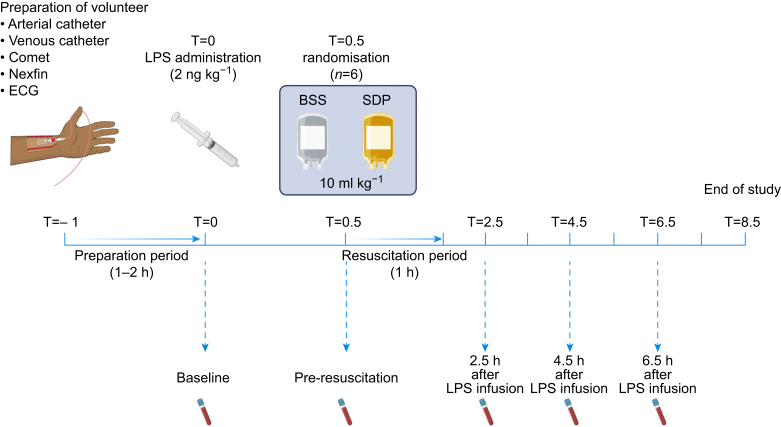
Study design and measurements. Volunteers (*n*=12) were admitted to the research unit of the intensive care department of the Amsterdam UMC. During the preparation period, all devices were installed and connected to monitor each subject and carry out study measurements. Thereafter, baseline samples were retrieved and 2 ng kg^−1^ lipopolysaccharide (LPS) from *Escherichia coli O113* (National Institute of Health Clinical Center, Bethesda, MD, USA) was injected to induce endotoxaemia (T=0). Thirty minutes after LPS administration (T=0.5), 10 ml kg^−1^ balanced salt solution (BSS) (*n*=6) (Plasma-Lyte 148, Baxter, Deerfield, IL, USA) or 10 ml kg^−1^ allogeneic Solvent Detergent Plasma (SDP) (*n*=6) (Omniplasma, Octapharma GmbH, Lachen, Switzerland) was infused in 1 h during the resuscitation period. Arterial blood was collected at baseline before LPS administration (T=0), 30 min after infusion of LPS but before transfusion (T=0.5), and 2.5, 4.5, and 6.5 h after infusion of LPS (T=2.5, T=4.5, and T=6.5). The experiment concluded 8.5 h after the LPS infusion, by which time all symptoms had subsided.

Self-reported symptoms were recorded hourly on a five-point severity scale.^29^ Arterial blood was collected at baseline before LPS administration, 30 min after infusion of LPS before transfusion, and 2.5, 4.5, and 6.5 h after starting the infusion of LPS (Fig. 1). Blood-gas variables were measured unadjusted for temperature (RapidLab 1265, Siemens, Munich, Germany) in lithium heparin anticoagulated blood. Complete blood counts and coagulation parameters (prothrombin time, fibrinogen, D-dimer, antithrombin) were measured in the central diagnostic laboratory of the Amsterdam UMC. All remaining blood samples were centrifuged twice to collect plasma (5804 R, Eppendorf AG, Hamburg, Germany), aliquoted, snap frozen in liquid nitrogen, and stored at −80°C until further evaluation.

### Biomarker measurement

Plasma biomarkers (see Supplementary material for full list) were measured in ethylenediaminetetraacetic acid (EDTA) anticoagulated blood at every time point using Luminex multiplex assay (R&D Systems, Minneapolis, MN, USA) read on a Bio-Plex 200 (BioRad, Hercules, CA, USA). Any measurement with less than 50 beads counted was judged to be unreliable and was therefore excluded for analysis. Interleukins-6 and -8 (IL-6 and IL-8), tumour necrosis factor alpha, thrombin–antithrombin complex (TATC) (all Abcam, Cambridge, UK), heparan sulphate (Elabscience, Houston, TX, USA), and chondroitin sulphate (Lifespan Biosciences, Lynnwood, WA, USA) were measured using enzyme-linked immunosorbent assays (ELISAs) (Supplementary material). Markers of NET formation (citrullinated histone H3 [Cayman Chemicals, Ann Arbor, MI, USA] and ELA2 [R&D Systems]) were measured in plasma samples using ELISAs (Supplementary material). ELISA values that were lower than the reference value were set at the lowest detection range. Human ADAMTS-13 antigen was measured using an ELISA technique and human ADAMTS-13 activity was measured using the FRETS-VWF73 assay.^30^^,^^31^

### Sample size calculation

Based on the syndecan-1 plasma levels in our pilot experiments comparing saline with plasma resuscitation in rats with endotoxaemia, a sample size of four subjects per group was calculated (*α*=0.05, *β*=0.2). To account for the lower predicted levels of syndecan-1 in this human model, which uses a far lower LPS dose, we included *n*=6 subjects per group.

### Statistical analysis

All data were regarded as non-parametric and are shown as medians with interquartile ranges (IQRs). Baseline differences between groups were calculated using the Mann–Whitney *U* test. To assess within-group changes over time, the Wilcoxon ranked sums test was used to compare values at T=0 with the maximum or minimum values observed during the study period. To analyse differences in continuous variables between groups over time, an aligned rank transform (ART) analysis of variance (anova) was performed, incorporating time point and group as fixed effects and subject ID as a random effect to account for repeated measures. Missing values were excluded on a per-marker basis. Type III Wald *F*-tests with Kenward–Roger degrees of freedom were used to evaluate statistical significance. A *P*-value of <0.05 was considered statistically significant. Mann–Whitney *U* tests and Wilcoxon ranked sum tests were performed in IBM SPSS statistics version 28 (IBM, Armonk, NY, USA). ART anova tests were conducted using the R packages ARTool and dplyr (Rstudio, version 4.4.3; R Core Team, 2024, Boston, MA, USA) and presented as graphs with GraphPad Prism 9 (GraphPad Software, San Diego, CA, USA).

## Results

### Study participants

Between April 2023 and October 2023, 24 male volunteers, ages 18–35 yr old, were assessed for eligibility, of whom 15 underwent screening. One was found ineligible because of abnormalities during screening. Additionally, two eligible participants withdrew from the study after screening before the scheduled appointment day (Supplementary Fig. 2). All participants who received LPS were included in the final analysis (Table 1). There were no serious adverse events.

**Table 1:** Participant characteristics. Variables were measured during screening. Data are presented as mean (sd) or as median (25th percentile–75th percentile).

Variable	Balanced salt solution	Solvent detergent plasma
	(*n*=6)	(*n*=6)
Age (yr)	26 (21–29)	22 (19–30)
Body mass index (kg m^−2^)	22.6 (1.3)	23.1 (1.0)
Heart rate (beats min^−1^)	67 (10.4)	73 (10.5)
Mean arterial blood pressure (mm Hg)	97 (92–104)	87 (83–100)
Haemoglobin (mmol L^−1^)	9.1 (8.9–9.6)	9.8 (9.2–10.2)
Leucocyte count (∗10^9^ L^−1^)	5.8 (1.3)	5.8 (1.4)
Platelet count (∗10^9^ L^−1^)	217 (35)	265 (30)

### Endotoxaemia model

Participants exhibited a typical response to infusion of low-dose LPS (Supplementary Figs 1 and 3),^29^^,^^32^ with median body temperature increasing from 37.0°C (IQR: 36.9–37.2°C) to 38.4°C (37.7–38.7°C). All participants met the criteria for systemic inflammatory response syndrome (SIRS).^33^

### Markers of systemic inflammation

**Fig 2:**
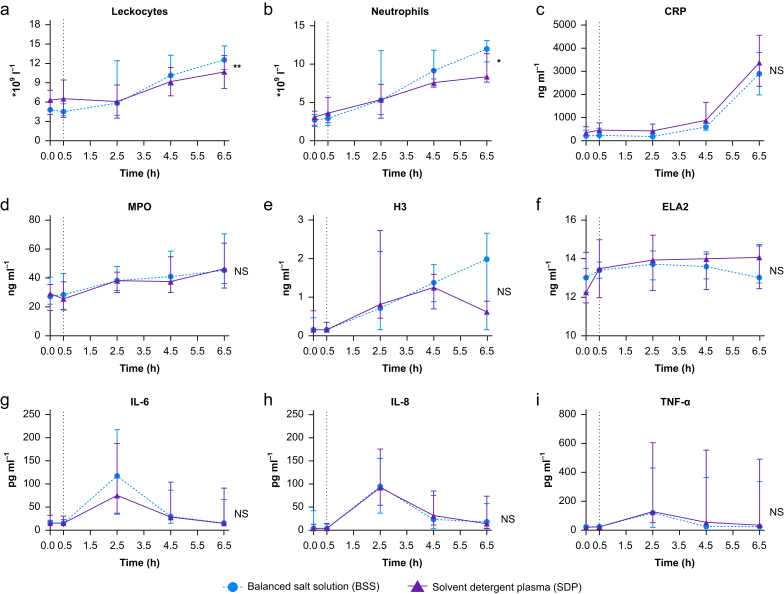
Effect of solvent detergent plasma and balanced salt solution on markers of inflammation and neutrophil extracellular trap formation. Lipopolysaccharide was given to all volunteers at T=0; the vertical dashed line represents the time point resuscitation was started. Data are presented as means with bars representing standard deviations. CRP, C-reactive protein; ELA2, neutrophil elastase 2; H3, citrullinated histone H3; IL, interleukin; MMP, matrix metalloproteinase; MPO, myeloperoxidase; NS, not significant; TNF-α, tumour necrosis factor alpha. ∗*P*<0.05; ∗∗*P*<0.01.

Leucocyte counts increased in parallel with increases in plasma IL-6 and IL-8 concentrations (Fig. 2). Neutrophil counts increased in both SDP and BSS groups after LPS injection, but to a lesser extent in participants receiving SDP, compared with those receiving BSS (Fig. 2b). Plasma cytokines and CRP levels increased in response to LPS, but did not differ between SDP and BSS (Fig. 2c–f).

### Neutrophil extracellular trap formation

Both MPO and H3 levels increased after LPS was infused, whereas ELA2 levels did not change. No differences were seen between SDP and BSS for any marker of NETosis (Fig. 2g–i).

### Glycocalyx degradation

**Fig 3:**
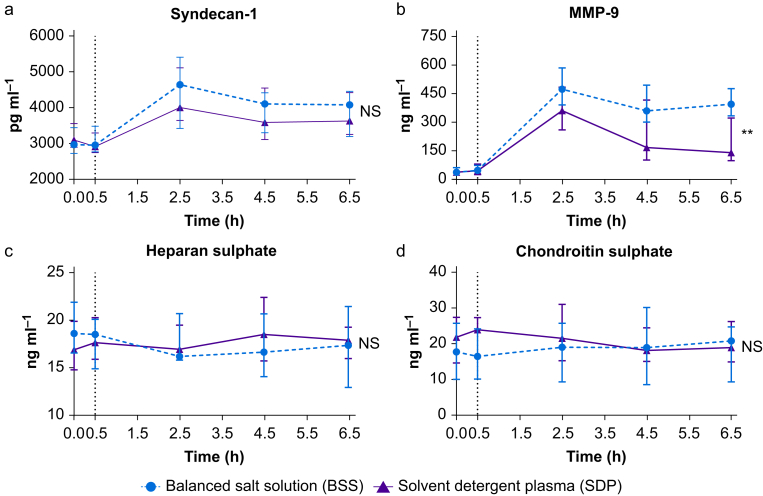
Effect of solvent detergent plasma and balanced salt solution on markers of glycocalyx degradation. Lipopolysaccharide was given to all volunteers at T=0, the vertical dashed line represents the time point resuscitation was started. Data are presented as means with bars representing standard deviations. MMP-9, matrix metalloproteinase 9; NS, not significant. ∗∗*P*<0.01.

Compared with baseline, syndecan-1 levels increased to the same extent in participants receiving BSS compared with SDP (Fig. 3a). MMP-9 levels were lower after LPS infusion with SDP treatment, compared with BSS (Fig. 3b). Other markers of glycocalyx degradation, including heparan sulphate and chondroitin sulphate, did not increase after LPS and did not differ between SDP and BSS (Fig. 3c and d).

### Endothelial injury and activation

**Fig 4:**
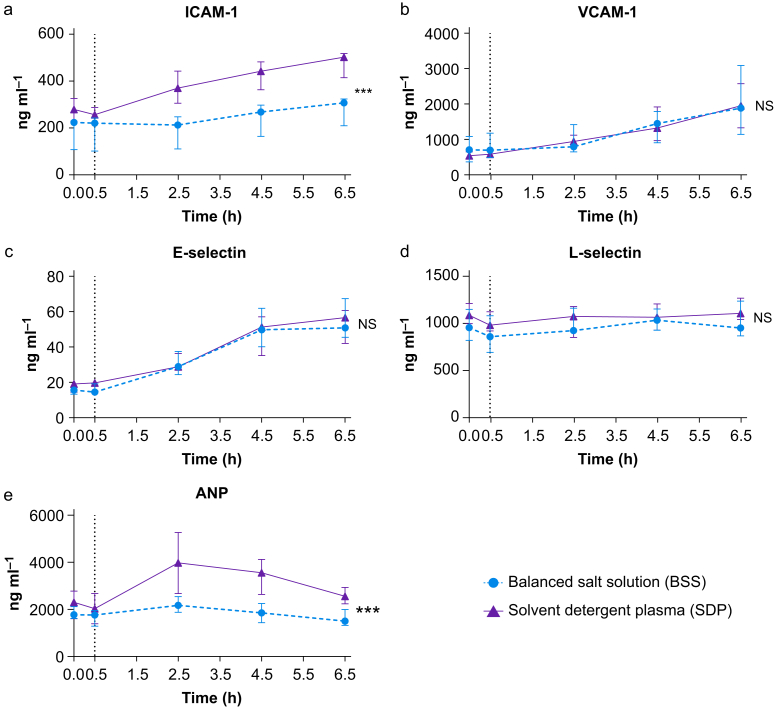
Effect of solvent detergent plasma and balanced salt solution on markers of endothelial injury and activation. Lipopolysaccharide was given to all volunteers at T=0, the vertical dashed line represents the time point resuscitation was started. Data are presented as means with bars representing standard deviations. ANP, atrial natriuretic peptide; ICAM-1, intercellular adhesion molecule 1; NS, not significant; VCAM-1, vascular cell adhesion protein 1. ∗∗∗*P*<0.001.

LPS resulted in endothelial activation, as indicated by increased levels of intercellular adhesion molecule 1 (ICAM-1), vascular cell adhesion protein 1 (VCAM-1), and E-selectin, compared with baseline (Fig. 4a–c). SDP transfusion resulted in higher ICAM-1 levels, compared with participants receiving BSS (Fig. 4a). LPS did not alter L-selectin concentrations (Fig. 4d). Atrial natriuretic peptide (ANP), a marker of vascular stretch, was elevated only in participants receiving SDP (Fig. 4e).

### Markers of coagulation

**Fig 5:**
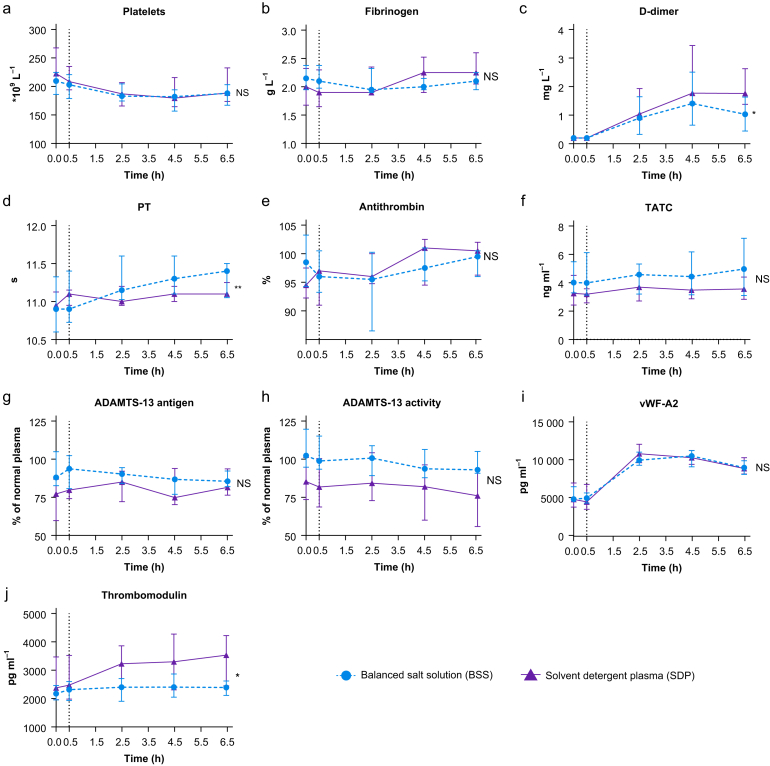
Effect of solvent detergent plasma and balanced salt solution on markers of coagulation. Lipopolysaccharide was given to all volunteers at T=0, the vertical dashed line represents the time point resuscitation was started. Data are presented as means with bars representing standard deviations. ADAMTS-13, a disintegrin and metalloproteinase with a thrombospondin type 1 motif, member 13; PT, prothrombin time; TATC, thrombin–antithrombin complex; vWF, von Willebrand factor. ∗*P*<0.05; ∗∗*P*<0.01.

Endotoxaemia led to a decrease in platelets and an increase in D-dimer, whereas fibrinogen levels and prothrombin time did not change during the experiment (Fig. 5a–d). In participants receiving SDP, prothrombin time was lower, and D-dimer levels were higher, compared with participants receiving BSS. Antithrombin and TATC levels did not change in response to LPS and remained comparable between SDP and BSS (Fig. 5e and f). Thrombomodulin levels were increased in participants receiving SDP and were higher compared with participants receiving BSS (Fig. 5j). LPS infusion led to vWF release in both groups. No effect on ADAMTS-13 antigen and activity was seen (Fig. 5g–i).

## Discussion

In this study, we found that SDP reduced immune activation, as indicated by lower neutrophil and leucocyte counts compared with BSS, resulting in a concomitant reduction of MMP-9, which mediates syndecan-1 shedding. Additionally, we found that plasma was associated with less PT prolongation, with increased D-dimer levels, but markers of thrombin generation remained unaffected.

LPS induced an inflammatory response, consistent with endotoxaemia, with activation of the endothelium and coagulation derangements, resembling mild endothelial injury seen in sepsis.^5^ Although SDP, compared with BSS, reduced leucocyte counts, neutrophil counts, and levels of MMP-9, an enzyme that mediates syndecan-1 degradation,^34^ it did not result in a significant decrease in levels of syndecan-1. An explanation for these findings may be that glycocalyx degradation was mild, given that LPS did not result in increased levels of other glycocalyx markers, such as heparan sulphate and chondroitin sulphate. Alternatively, the effects of plasma on preservation of the glycocalyx may be less significant than hypothesised. In a previous substudy of an RCT, we demonstrated that plasma reduced syndecan-1 levels in septic patients, suggesting endothelial protective properties of plasma.^25^ Of note, the severity of endothelial injury observed in this study was significantly higher compared with the current study. In contrast, a recent RCT comparing plasma with Ringer-acetate resuscitation in patients with septic shock found no differences in syndecan-1 or E-selectin between groups.^35^ However, baseline differences and the receipt of large volumes of crystalloids before randomisation hamper conclusions drawn from this trial.

Our data contrast with findings from animal models of septic and traumatic shock, in which plasma resuscitation reduced syndecan-1 levels compared with crystalloid resuscitation.^10^^,^^19^^,^^21^, ^22^, ^23^, ^24^ In all these models, the benefits of plasma were associated with a threefold reduction in volume needed for resuscitation. In a sepsis animal model where similar volumes were transfused, plasma did not reduce glycocalyx injury.^20^ This finding is similar to the recent RCT in patients with sepsis, which also used comparable volumes of plasma.^35^ If the volume in the BSS group of our model had been higher to reach adequate intravascular expansion, potential positive effects of plasma might have been more pronounced. However, this would have required adding an additional group in which participants received approximately three times more BSS for resuscitation, a group we did not include for safety reasons. Alternatively, the glycocalyx preserving effect of plasma may depend on the presence of shock. Our model is not a shock model, as tissue perfusion remained adequate, as reflected by low lactate levels throughout the experiment. Thereby, the question of whether plasma can reduce glycocalyx degradation in patients with shock by limiting resuscitation volume to meet hemodynamic goals remains unanswered.

Notably, an increase in ANP was observed only in the SDP group. Because of its high protein content, plasma more effectively increases colloid osmotic pressure, resulting in a greater increase in intravascular volume compared with the BSS group. The participants in this study were normovolaemic before LPS administration. Therefore, the amount of SDP in this study likely led to an increase in transfusion-associated circulatory overload biomarkers, further underlined by higher thrombomodulin and ICAM-1 levels found in the plasma group, suggesting vascular stretch. Both thrombomodulin and ICAM-1 have been shown to be elevated when endothelial cells are exposed to strain, which occurs during volume overload.^36^, ^37^, ^38^ As participants were young and likely able to adequately compensate for the administered fluid volume, they exhibited no signs of fluid overload.

Plasma is primarily administered to correct coagulopathy by replenishing deficits of coagulation factors. In the current study, LPS resulted in a slight PT prolongation and increased D-dimer levels, consistent with septic coagulopathy. Plasma was associated with inhibited PT prolongation, probably by supplementing coagulation factors. Notably, there was a slight increase in D-dimer levels among those receiving SDP. Although we were not able to measure thrombin generation directly, TATC, as a marker of thrombin generation, remained unaltered. Therefore, we do not think that this D-dimer increase reflects augmentation of an already heightened thrombin generation. Also, there were no differences in vWF secretion between groups. Taken together, we suggest that plasma transfusion during a septic coagulopathy response does not ‘add fuel to the fire’, but rather limits the coagulation response that is typically observed in sepsis. In line with this, patients with septic shock receiving plasma had similar Disseminated Intravascular Coagulation (DIC) scores compared with those receiving Ringer-acetate.^35^

Our study has some limitations. First, although LPS-induced endotoxaemia triggers a systemic inflammatory response that meets the criteria for SIRS and resembles that seen in sepsis,^29^ the pathophysiology of endotoxaemia differs significantly from that of sepsis and septic shock. In particular, the degrees of endothelial injury, dysregulated inflammation, and coagulopathy are considerably lower in endotoxaemia than in sepsis. Although endotoxaemia models are valuable for advancing our understanding of the complex pathophysiologic mechanisms underlying sepsis and for exploring potential therapeutic interventions, the findings from this study cannot be directly extrapolated to patients with sepsis or septic shock.^29^ Moreover, we only included male participants, which limits the generalisability to females, considering that females typically exhibit a more pronounced inflammatory response to LPS treatment.^39^ However, as we found no differences in immune response between groups, it is unlikely that we would have observed significant differences had we included females. To add to this, the investigated population consisted of young, healthy adults. Our results might differ compared with an older population with comorbidities. However, this was a deliberate choice made for safety reasons concerning the volunteers. Lastly, SDP is not the standard plasma product used globally, which may limit the generalisability of our findings. However, because SDP is a pooled product, variability in components such as cytokines levels is minimised, likely resulting in a more homogenous effect.

In summary, in a human endotoxaemia model, SDP resuscitation reduced leucocyte and neutrophil levels, resulting in a concomitant reduction of MMP-9. Endothelial injury and glycocalyx degradation were not altered by plasma, compared with resuscitation with an equal volume of BSS. Further research is needed to determine whether plasma has a role in shock resuscitation during sepsis.

## Authors’ contributions

Conceptualisation: DvdB, DK, PS

Data curation: DvdB, BH

Formal analysis: DvdB, DK

Funding acquisition: NJ

Investigation: DvdB, BH, IP, KV

Methodology: DvdB, BH, DK, RK, KV, PS, NJ

Project administration: DvdB, NJ

Resources: KV, NJ

Supervision: DvdB, DK, NJ

Validation: DvdB, BH, IP

Visualisation: DvdB, DK

Writing—original draft: DvdB, NJ

Writing—review and editing: all authors

## Funding

Octapharma BV.

## Declaration of interest

NPJ has received research support from Octapharma BV and Werfen BV and is a consultant at Bayer BV. The other authors declare that they have no conflicts of interest.

## References

[1] Papayannopoulos V. (2018). Neutrophil extracellular traps in immunity and disease. Nat Rev Immunol.

[2] Ma Y., Yang X., Chatterjee V., Meegan J.E., Beard R.S., Yuan S.Y. (2019). Role of neutrophil extracellular traps and vesicles in regulating vascular endothelial permeability. Front Immunol.

[3] Patterson E.K., Cepinskas G., Fraser D.D. (2022). Endothelial glycocalyx degradation in critical illness and injury. Front Med (Lausanne).

[4] Johansson P., Stensballe J., Ostrowski S. (2017). Shock induced endotheliopathy (SHINE) in acute critical illness – a unifying pathophysiologic mechanism. Crit Care.

[5] Ince C., Mayeux P.R., Nguyen T. (2016). The endothelium in sepsis. Shock.

[6] Evans L., Rhodes A., Alhazzani W. (2021). Surviving sepsis campaign: international guidelines for management of sepsis and septic shock 2021. Intensive Care Med.

[7] Arabi Y.M., Belley-Cote E., Carsetti A. (2024). European Society of Intensive Care Medicine clinical practice guideline on fluid therapy in adult critically ill patients. Part 1: the choice of resuscitation fluids. Intensive Care Med.

[8] Zeng Y., Adamson R.H., Curry F.R., Tarbell J.M. (2014). Sphingosine-1-phosphate protects endothelial glycocalyx by inhibiting syndecan-1 shedding. Am J Physiol Heart Circ Physiol.

[9] Cheung-Flynn J., Alvis B.D., Hocking K.M. (2019). Normal saline solutions cause endothelial dysfunction through loss of membrane integrity, ATP release, and inflammatory responses mediated by P2X7R/p38 MAPK/MK2 signaling pathways. PLoS One.

[10] Torres L.N., Chung K.K., Salgado C.L., Dubick M.A., Torres Filho I.P. (2017). Low-volume resuscitation with normal saline is associated with microvascular endothelial dysfunction after hemorrhage in rats, compared to colloids and balanced crystalloids. Crit Care.

[11] Kremer H., Baron-Menguy C., Tesse A. (2011). Human serum albumin improves endothelial dysfunction and survival during experimental endotoxemia: concentration-dependent properties. Crit Care Med.

[12] Finfer S., Bellomo R., Boyce N. (2004). A comparison of albumin and saline for fluid resuscitation in the intensive care unit. N Engl J Med.

[13] Caironi P., Tognoni G., Masson S. (2014). Albumin replacement in patients with severe sepsis or septic shock. N Engl J Med.

[14] Finfer S., Bellomo R., McEvoy S. (2006). Effect of baseline serum albumin concentration on outcome of resuscitation with albumin or saline in patients in intensive care units: analysis of data from the saline versus albumin fluid evaluation (SAFE) study. BMJ.

[15] van den Brink D.P., Kleinveld D.J.B., Sloos P.H. (2021). Plasma as a resuscitation fluid for volume-depleted shock: Potential benefits and risks. Transfusion.

[16] Milford E.M., Reade M.C. (2019). Resuscitation fluid choices to preserve the endothelial glycocalyx. Crit Care.

[17] Levi M., Scully M., Singer M. (2018). The role of ADAMTS-13 in the coagulopathy of sepsis. J Thromb Haemost.

[18] Kleinveld D.J.B., Simons D.D.G., Dekimpe C. (2021). Plasma and rhADAMTS13 reduce trauma-induced organ failure by restoring the ADAMTS13-VWF axis. Blood Adv.

[19] Chang R., Holcomb J.B., Johansson P.I., Pati S., Schreiber M.A., Wade C.E. (2018). Plasma resuscitation improved survival in a cecal ligation and puncture rat model of sepsis. Shock.

[20] van den Brink D.P., Kleinveld D.J.B., Bongers A. (2023). The effects of resuscitation with different plasma products on endothelial permeability and organ injury in a rat pneumosepsis model. Intensive Care Med Exp.

[21] Kozar R.A., Peng Z., Zhang R. (2011). Plasma restoration of endothelial glycocalyx in a rodent model of hemorrhagic shock. Anesth Analg.

[22] Peng Z., Pati S., Potter D. (2013). Fresh frozen plasma lessens pulmonary endothelial inflammation and hyperpermeability after hemorrhagic shock and is associated with loss of syndecan 1. Shock.

[23] Torres L.N., Sondeen J.L., Ji L., Dubick M.A., Torres Filho I. (2013). Evaluation of resuscitation fluids on endothelial glycocalyx, venular blood flow, and coagulation function after hemorrhagic shock in rats. J Trauma Acute Care Surg.

[24] Torres Filho I.P., Torres L.N., Salgado C., Dubick M.A. (2016). Plasma syndecan-1 and heparan sulfate correlate with microvascular glycocalyx degradation in hemorrhaged rats after different resuscitation fluids. Am J Physiol Heart Circ Physiol.

[25] Straat M., Müller M.C., Meijers J.C. (2015). Effect of transfusion of fresh frozen plasma on parameters of endothelial condition and inflammatory status in non-bleeding critically ill patients: a prospective substudy of a randomized trial. Crit Care.

[26] Gruen D.S., Brown J.B., Guyette F.X. (2020). Prehospital plasma is associated with distinct biomarker expression following injury. JCI Insight.

[27] Schulz K.F., Altman D.G., Moher D. (2010). CONSORT 2010 statement: updated guidelines for reporting parallel group randomised trials. BMJ.

[28] Urbaniak, G. C., & Plous, S. (2013). Research Randomizer (Version 4.0) [Computer program] [cited 01-07-2023]. http://www.randomizer.org/

[29] van Lier D., Geven C., Leijte G.P., Pickkers P. (2019). Experimental human endotoxemia as a model of systemic inflammation. Biochimie.

[30] Dekimpe C., Roose E., Tersteeg C. (2020). Anti-ADAMTS13 autoantibodies in immune-mediated thrombotic thrombocytopenic purpura do not hamper ELISA-based quantification of ADAMTS13 antigen. J Thromb Haemost.

[31] Schelpe A.S., Orlando C., Ercig B. (2018). Child-onset thrombotic thrombocytopenic purpura caused by p.R498C and p.G259PfsX133 mutations in ADAMTS13. Eur J Haematol.

[32] Fullerton J.N., Segre E., De Maeyer R.P., Maini A.A., Gilroy D.W. (2016). Intravenous endotoxin challenge in healthy humans: an experimental platform to investigate and modulate systemic inflammation. J Vis Exp.

[33] Singer M., Deutschman C.S., Seymour C.W. (2016). The Third International Consensus Definitions for Sepsis and Septic Shock (Sepsis-3). JAMA.

[34] Zhang D., Zhang J.T., Pan Y. (2021). Syndecan-1 shedding by matrix metalloproteinase-9 signaling regulates alveolar epithelial tight junction in lipopolysaccharide-induced early acute lung injury. J Inflamm Res.

[35] Clausen N.E., Meyhoff C.S., Henriksen H.H. (2024). Plasma as endothelial rescue in septic shock: a randomized, phase 2a pilot trial. Transfusion.

[36] Martin F.A., McLoughlin A., Rochfort K.D., Davenport C., Murphy R.P., Cummins P.M. (2014). Regulation of thrombomodulin expression and release in human aortic endothelial cells by cyclic strain. PLoS One.

[37] Sung H.J., Yee A., Eskin S.G., McIntire L.V. (2007). Cyclic strain and motion control produce opposite oxidative responses in two human endothelial cell types. Am J Physiol Cell Physiol.

[38] Salvador A.M., Nevers T., Velázquez F. (2016). Intercellular adhesion molecule 1 regulates left ventricular leukocyte infiltration, cardiac remodeling, and function in pressure overload-induced heart failure. J Am Heart Assoc.

[39] Wegner A., Elsenbruch S., Rebernik L. (2015). Inflammation-induced pain sensitization in men and women: does sex matter in experimental endotoxemia?. Pain.

