# Supplementary material for: The effects of balanced crystalloid versus plasma on endothelial injury, systemic inflammation, and coagulation in experimental endotoxaemia: a randomised human volunteer study
Source: Br J Anaesth. 2025 Oct 18;136(1):343–52. doi: 10.1016/j.bja.2025.08.060 (PMC12851875; doi:10.1016/j.bja.2025.08.060)
Supplement: Multimedia component 1 [file mmc1.docx]

**The effects of balanced crystalloid versus plasma on endothelial injury, systemic inflammation and coagulation in experimental endotoxaemia: a randomized human volunteer study.**

Daan P. van den Brink et al

**SUPPLEMENTARY DATA**

Contents

[CONSORT 2010 checklist of information 2](#_Toc209033463)

[Supplementary Table 1: Study inclusion and exclusion criteria. 4](#_Toc209033464)

[Supplementary Figure 1: CONSORT diagram 4](#_Toc209033465)

[Supplementary Figure 2: Physiological response to LPS. 6](#_Toc209033466)

[Supplementary Figure 3: LPS-related symptoms. 7](#_Toc209033467)

[Supplementary methods: LUMINEX standard curves. 8](#_Toc209033468)

[CRP 8](#_Toc209033469)

[L-selectin 8](#_Toc209033470)

[MMP-9 9](#_Toc209033471)

[MMP-2: 9](#_Toc209033472)

[MPO 10](#_Toc209033473)

[ANP 10](#_Toc209033474)

[E-selectin 11](#_Toc209033475)

[Thrombomodulin 11](#_Toc209033476)

[VCAM-1 12](#_Toc209033477)

[ICAM-1 12](#_Toc209033478)

[Syndecan-1 13](#_Toc209033479)

[Supplementary methods: ELISA standard curves. 14](#_Toc209033480)

[Standard curve heparan sulphate 14](#_Toc209033481)

[Standard curve Chondroitin sulphate 15](#_Toc209033482)

[Standard curve H3 16](#_Toc209033483)

[Standard curve ELA2 17](#_Toc209033484)

[Standard curve IL-6 18](#_Toc209033485)

[Standard curve IL-8 19](#_Toc209033486)

[Standard curve TNF-a 20](#_Toc209033487)

[Standard curve TATC 21](#_Toc209033488)


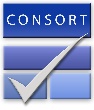
CONSORT 2010 checklist of information

| Section/Topic | Item No | Checklist item | Reported on page No |
| --- | --- | --- | --- |
| Title and abstract | | | |
|  | 1a | Identification as a randomised trial in the title | 1 |
|  | 1b | Structured summary of trial design, methods, results, and conclusions (for specific guidance see CONSORT for abstracts) | 2 |
| Introduction | | | |
| Background and objectives | 2a | Scientific background and explanation of rationale | 3-4 |
|  | 2b | Specific objectives or hypotheses | 4 |
| Methods | | | |
| Trial design | 3a | Description of trial design (such as parallel, factorial) including allocation ratio | 5-6 |
|  | 3b | Important changes to methods after trial commencement (such as eligibility criteria), with reasons | 5-6 |
| Participants | 4a | Eligibility criteria for participants | 5  Table S1 |
|  | 4b | Settings and locations where the data were collected | 5 |
| Interventions | 5 | The interventions for each group with sufficient details to allow replication, including how and when they were actually administered | 5-6 |
| Outcomes | 6a | Completely defined pre-specified primary and secondary outcome measures, including how and when they were assessed | 6-7 |
|  | 6b | Any changes to trial outcomes after the trial commenced, with reasons | N.A. |
| Sample size | 7a | How sample size was determined | 7 |
|  | 7b | When applicable, explanation of any interim analyses and stopping guidelines | N.A. |
| Randomisation: |  |  |  |
| Sequence generation | 8a | Method used to generate the random allocation sequence | 5-6 |
|  | 8b | Type of randomisation; details of any restriction (such as blocking and block size) | 5-6 |
| Allocation concealment mechanism | 9 | Mechanism used to implement the random allocation sequence (such as sequentially numbered containers), describing any steps taken to conceal the sequence until interventions were assigned | 5-6 |
| Implementation | 10 | Who generated the random allocation sequence, who enrolled participants, and who assigned participants to interventions | 5-6 |
| Blinding | 11a | If done, who was blinded after assignment to interventions (for example, participants, care providers, those assessing outcomes) and how | 5-6 |
|  | 11b | If relevant, description of the similarity of interventions | N.A. |
| Statistical methods | 12a | Statistical methods used to compare groups for primary and secondary outcomes | 8 |
|  | 12b | Methods for additional analyses, such as subgroup analyses and adjusted analyses | 8 |
| Results | | | |
| Participant flow (a diagram is strongly recommended) | 13a | For each group, the numbers of participants who were randomly assigned, received intended treatment, and were analysed for the primary outcome | 9  Fig S2 |
|  | 13b | For each group, losses and exclusions after randomisation, together with reasons | Fig S2 |
| Recruitment | 14a | Dates defining the periods of recruitment and follow-up | 5 |
|  | 14b | Why the trial ended or was stopped | N.A. |
| Baseline data | 15 | A table showing baseline demographic and clinical characteristics for each group | Table 1 |
| Numbers analysed | 16 | For each group, number of participants (denominator) included in each analysis and whether the analysis was by original assigned groups | Figures |
| Outcomes and estimation | 17a | For each primary and secondary outcome, results for each group, and the estimated effect size and its precision (such as 95% confidence interval) | 9-11 |
|  | 17b | For binary outcomes, presentation of both absolute and relative effect sizes is recommended | 9-11 |
| Ancillary analyses | 18 | Results of any other analyses performed, including subgroup analyses and adjusted analyses, distinguishing pre-specified from exploratory | 9-11 |
| Harms | 19 | All important harms or unintended effects in each group (for specific guidance see CONSORT for harms) | N.A. |
| Discussion | | | |
| Limitations | 20 | Trial limitations, addressing sources of potential bias, imprecision, and, if relevant, multiplicity of analyses | 14 |
| Generalisability | 21 | Generalisability (external validity, applicability) of the trial findings | 12-14 |
| Interpretation | 22 | Interpretation consistent with results, balancing benefits and harms, and considering other relevant evidence | 12-14 |
| Other information | | |  |
| Registration | 23 | Registration number and name of trial registry | N.A. |
| Protocol | 24 | Where the full trial protocol can be accessed, if available | On request |
| Funding | 25 | Sources of funding and other support (such as supply of drugs), role of funders | 19 |

# Supplementary Table 1: Study inclusion and exclusion criteria.

| **Inclusion criteria:** |
| --- |
| - Healthy male volunteers  - 18-35 years of age  - BMI between 20-25. |
| **Exclusion criteria:** |
| - No informed consent  - Abnormal lab or urine test results during the screening prior to inclusion of the study  - Use of medication on prescription  - Given a blood donation < 3 months prior to study participation  - Received previous blood transfusion  - Participation in other medical study < 3months  - Participation in previous volunteer studies using LPS  - Fever at the intake or on the research day prior to LPS infusion |

# Supplementary Figure 1: CONSORT diagram

Study participant flow through enrollment, allocation, follow-up and analysis

Enrollment

Assessed for eligibility (n=24)

Excluded (n=10)

- Not meeting inclusion criteria during screening (5)

- Declined to participate (5)

Randomized (n=15)

Allocated to control (Balanced salt solution group) (n=8)

- Received allocated intervention (n=6)

- Did not receive allocated intervention due to study withdrawal between allocation and first study appointment (n=2)

Allocated to intervention (Solvent detergent plasma group) (n=7)

- Received allocated intervention (n=6)

- Did not receive allocated intervention due to abnormalities found in ecg during study day before LPS administration (n=1)

Allocation

Lost to follow-up (n=0)

Lost to follow-up (n=0)

Follow-Up

Analysed (n=6)

Analysed (n=6)

Analysis

# Supplementary Figure 2: Physiological response to LPS.

Lipopolysaccharide was given to all volunteers at T=0, the vertical dashed line represents the timepoint resuscitation was started. Data are presented as means with bars representing standard deviations. N.S. = not significant





# Supplementary Figure 3: LPS-related symptoms.


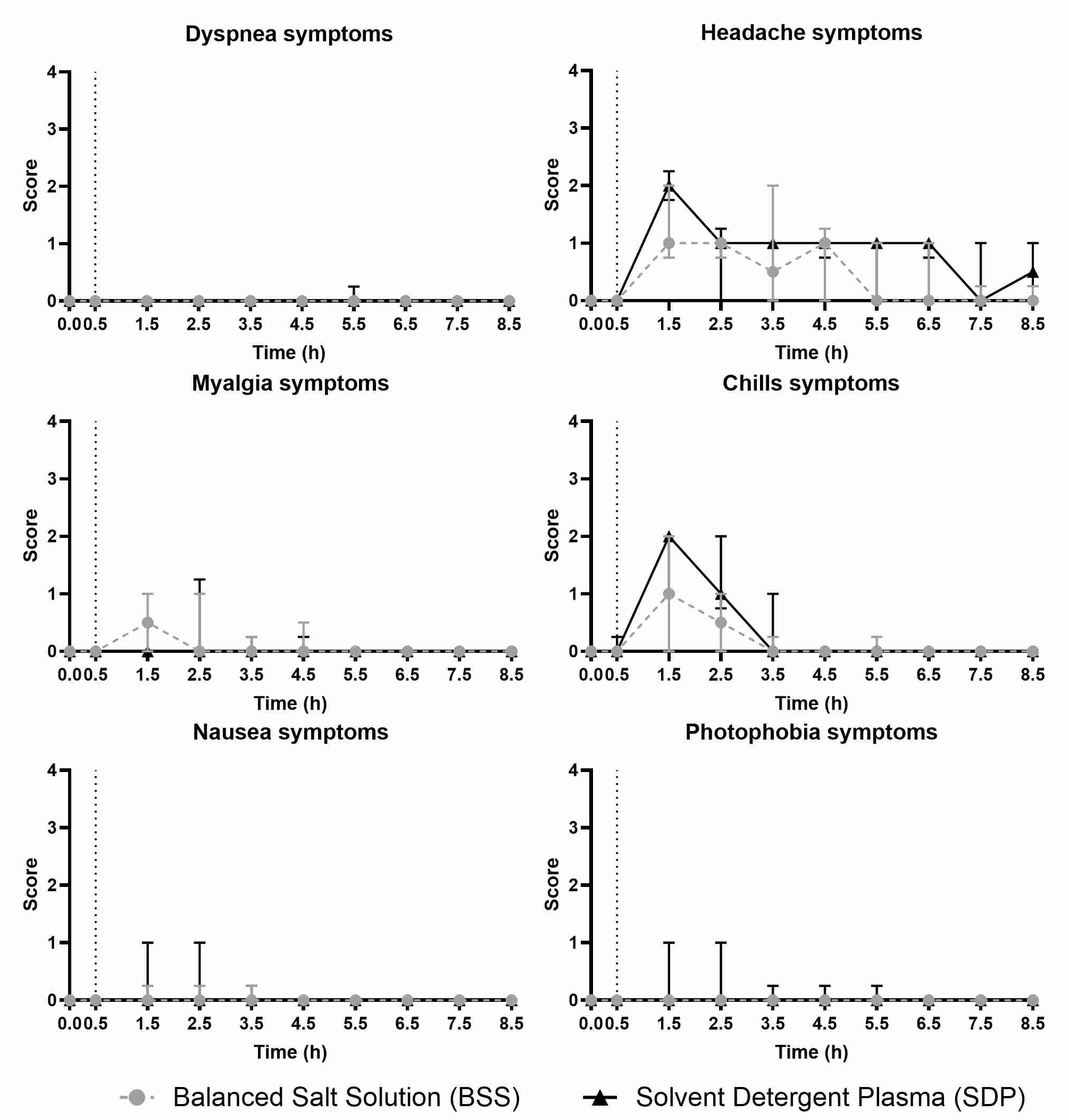


*Lipopolysaccharide was given to all volunteers at T=0, the vertical dashed line represents the timepoint resuscitation was started. Data are presented as median with bars presenting interquartile ranges.*  Symptoms were scored on a 5-point scale (mild – moderate – severe – life-threatening – fatal), if a symptom was absent a score of 0 was given.

# Supplementary methods: LUMINEX standard curves.

Plasma biomarkers syndecan-1, E-selectin, L-selectin, intercellular adhesion molecule 1 (ICAM-1), vascular cell adhesion protein 1 (VCAM-1), thrombomodulin, matrix metalloproteinase 2 (MMP-2), matrix metalloproteinase 9 (MMP-9), atrial natriuretic peptide (ANP), C-reactive protein (CRP), von Willebrand Factor (vWF) and myeloperoxidase (MPO) were measured.

## CRP

|    \|  \| \| --- \| |
| --- | --- |
|    \|  \| \| --- \| |
| Regression Type: Logistic - 5PL |
| Std. Curve: FI = -2,85105 + (4852,25 + 2,85105) / ((1 + (Conc / 13149,8)^-1,25111))^0,736021 |
| FitProb. = 0,7441, ResVar. = 0,5426 |

## L-selectin

|    \|  \| \| --- \| |
| --- | --- |
|    \|  \| \| --- \| |
| Regression Type: Logistic - 5PL |
| Std. Curve: FI = 1,2991 + (15029,4 - 1,2991) / ((1 + (Conc / 258206)^-0,880871))^2,17244 |
| FitProb. = 0,7310, ResVar. = 0,3133 |

## MMP-9

|    \|  \| \| --- \| |
| --- | --- |
|    \|  \| \| --- \| |
| Regression Type: Logistic - 5PL |
| Std. Curve: FI = 1,11561 + (6075,01 - 1,11561) / ((1 + (Conc / 26047,1)^-2,44074))^0,491888 |
| FitProb. = 0,9124, ResVar. = 0,2457 |

## MMP-2:

|    \|  \| \| --- \| |
| --- | --- |
|    \|  \| \| --- \| |
| Regression Type: Logistic - 5PL |
| Std. Curve: FI = 2,53221 + (6702,53 - 2,53221) / ((1 + (Conc / 15631,5)^-2,04759))^0,891297 |
| FitProb. = 0,5415, ResVar. = 0,6134 |

## MPO

|    \|  \| \| --- \| |
| --- | --- |
|    \|  \| \| --- \| |
| Regression Type: Logistic - 5PL |
| Std. Curve: FI = -0,136564 + (3377,15 + 0,136564) / ((1 + (Conc / 15066,9)^-2,1759))^0,444342 |
| FitProb. = 0,9801, ResVar. = 0,1071 |

## ANP

|    \|  \| \| --- \| |
| --- | --- |
|    \|  \| \| --- \| |
| Regression Type: Logistic - 5PL |
| Std. Curve: FI = 2,38059 + (3900,97 - 2,38059) / ((1 + (Conc / 3900,64)^-1,1179))^1,52246 |
| FitProb. = 0,2868, ResVar. = 1,2583 |

## E-selectin

|    \|  \| \| --- \| |
| --- | --- |
|    \|  \| \| --- \| |
| Regression Type: Logistic - 5PL |
| Std. Curve: FI = 0,588211 + (7451,08 - 0,588211) / ((1 + (Conc / 12093,5)^-1,0447))^0,995183 |
| FitProb. = 0,9463, ResVar. = 0,2370 |

## Thrombomodulin

|    \|  \| \| --- \| |
| --- | --- |
|    \|  \| \| --- \| |
| Regression Type: Logistic - 5PL |
| Std. Curve: FI = 2,49736 + (27830 - 2,49736) / ((1 + (Conc / 20785,3)^-0,51874))^2,14719 |
| FitProb. = 0,9655, ResVar. = 0,1444 |
|  |

## VCAM-1

|    \|  \| \| --- \| |
| --- | --- |
|    \|  \| \| --- \| |
| Regression Type: Logistic - 5PL |
| Std. Curve: FI = 0,662132 + (2052,4 - 0,662132) / ((1 + (Conc / 484667)^-0,881864))^1,41784 |
| FitProb. = 0,9739, ResVar. = 0,0742 |

## ICAM-1

|    \|  \| \| --- \| |
| --- | --- |
|    \|  \| \| --- \| |
| Regression Type: Logistic - 5PL |
| Std. Curve: FI = 1,2715 + (7706,03 - 1,2715) / ((1 + (Conc / 313744)^-1,76203))^0,690066 |
| FitProb. = 0,7517, ResVar. = 0,5326 |

## Syndecan-1

|    \|  \| \| --- \| |
| --- | --- |
|    \|  \| \| --- \| |
| Regression Type: Logistic - 5PL |
| Std. Curve: FI = 2,93957 + (4799,79 - 2,93957) / ((1 + (Conc / 20957,6)^-1,06028))^1,02197 |
| FitProb. = 0,9704, ResVar. = 0,0810 |

# Supplementary methods: ELISA standard curves.

## Standard curve heparan sulphate

**
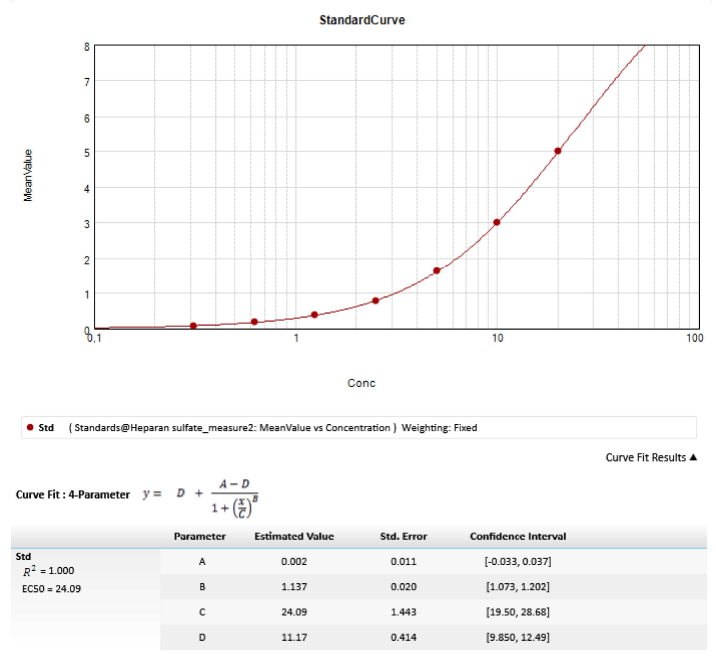
**

**
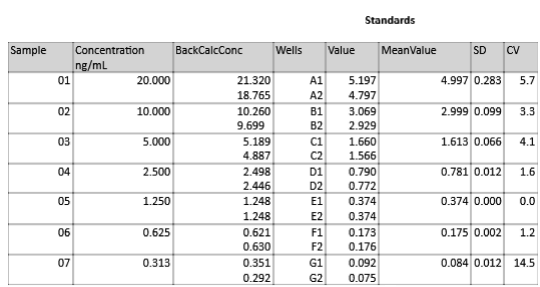
**

**Highest detection limit:** 20 ng/mL

**Lowest detection limit:** 0.31 ng/mL

## Standard curve Chondroitin sulphate

**
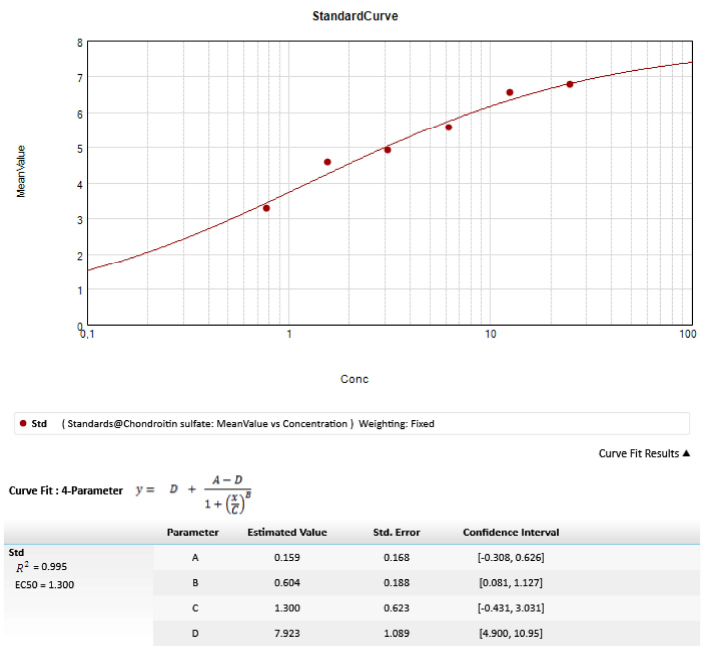
**

**
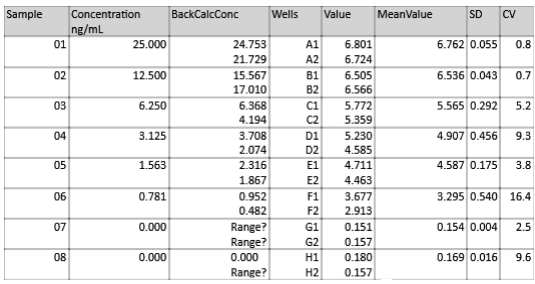
**

**Highest detection limit:** 25 ng/mL

**Lowest detection limit:** 0.40 ng/mL

## Standard curve H3

**
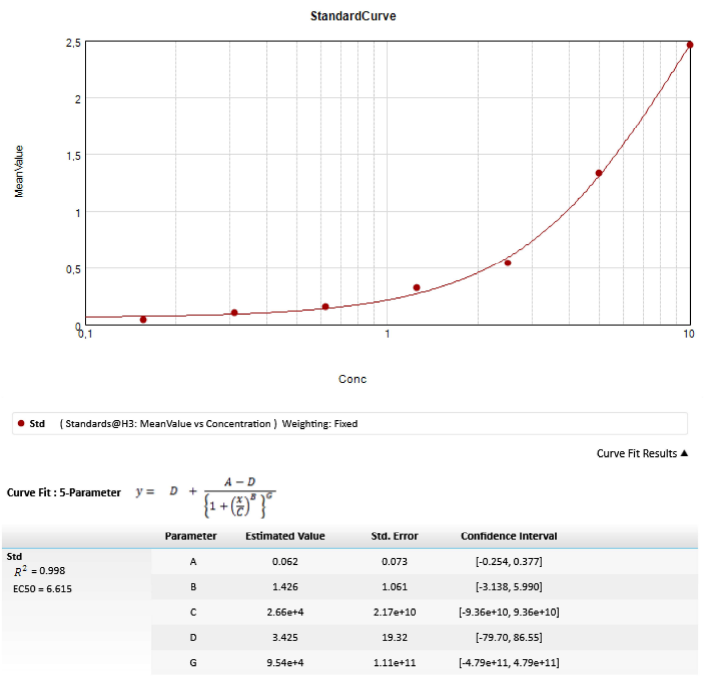
**

**
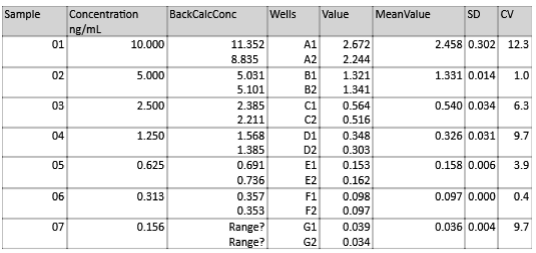
**

**Highest detection limit:** 10 ng/mL

**Lowest detection limit:** 0.156 ng/mL

## Standard curve ELA2

**
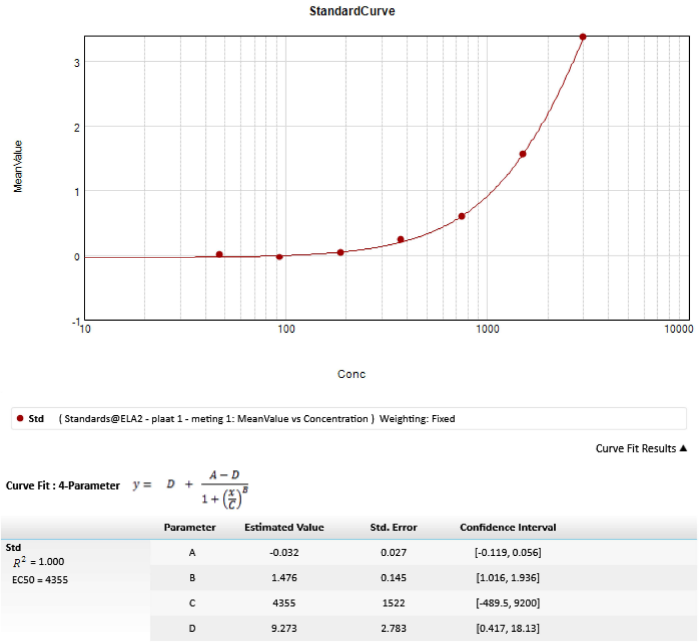
**


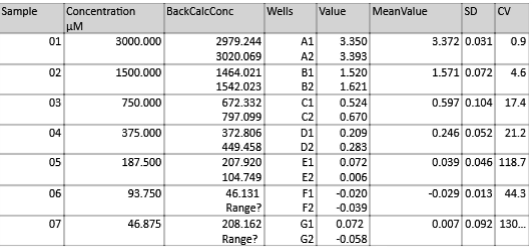


**Highest detection limit:** 3000 pg/mL

**Lowest detection limit:** 47 pg/mL

## Standard curve IL-6


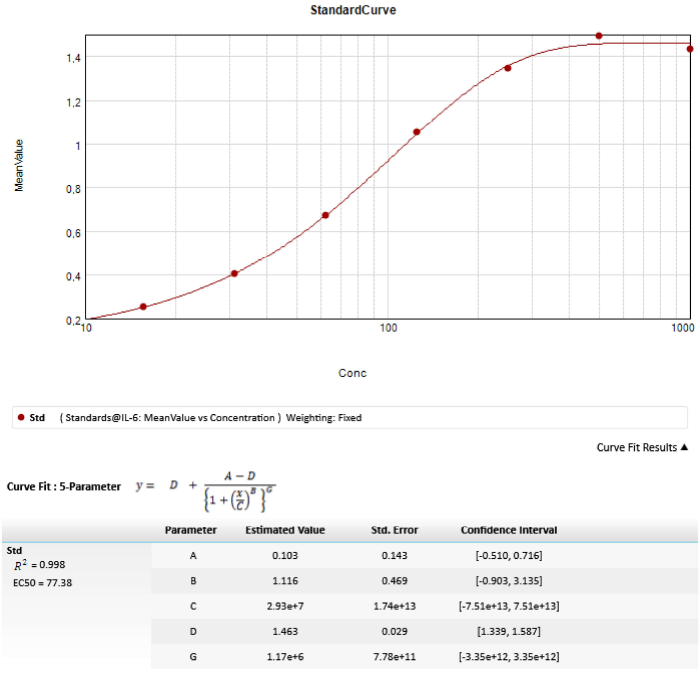


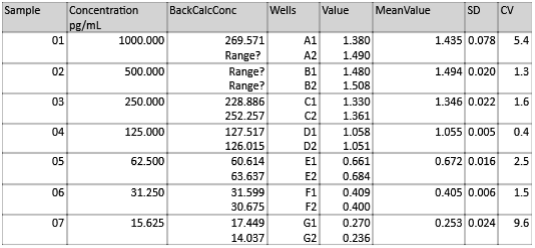


**Lowest detection limit:** 1000 pg/mL

**Highest detection limit**: 15 pg/mL

## Standard curve IL-8


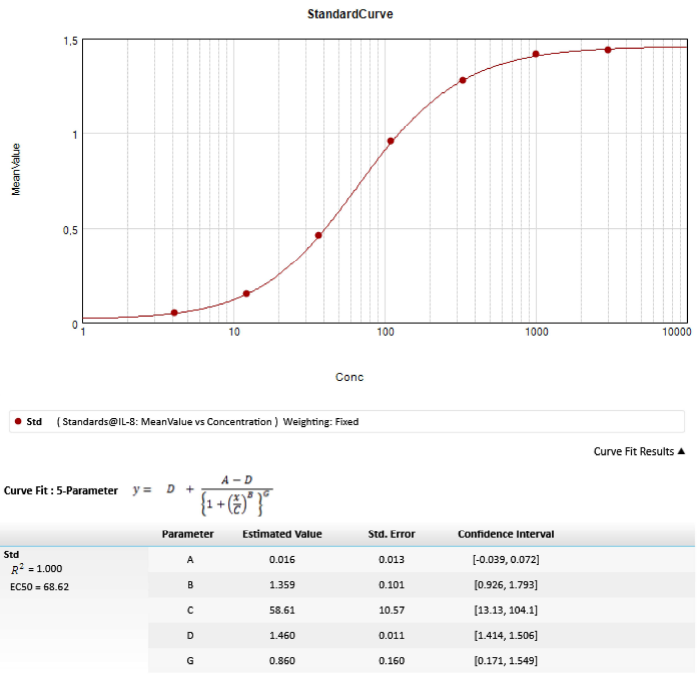


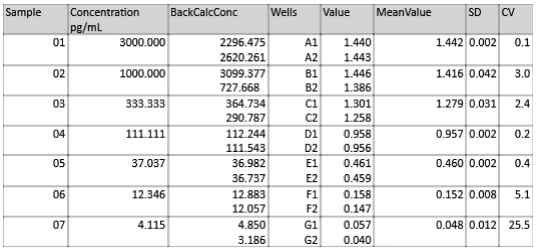


**Highest detection limit:** 3000 pg/mL

**Lowest detection limit**: 4 pg/mL

## Standard curve TNF-a

**
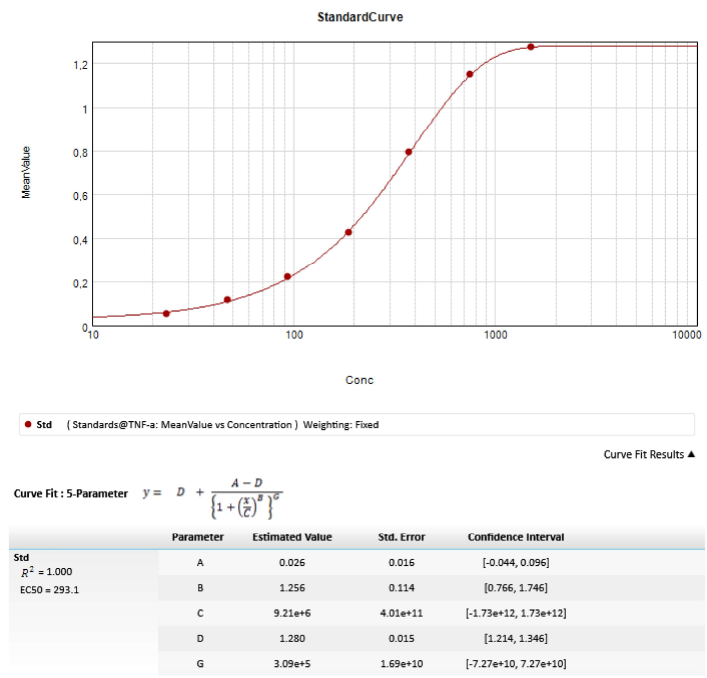
**


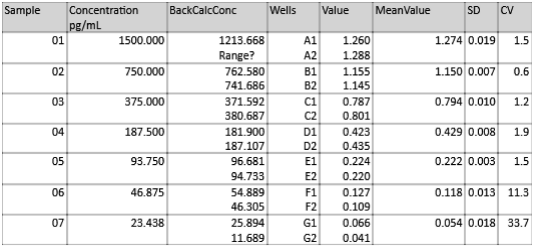


**Highest detection limit:** 1500 pg/mL

**Lowest detection limit**: 23 pg/mL

## Standard curve TATC

**
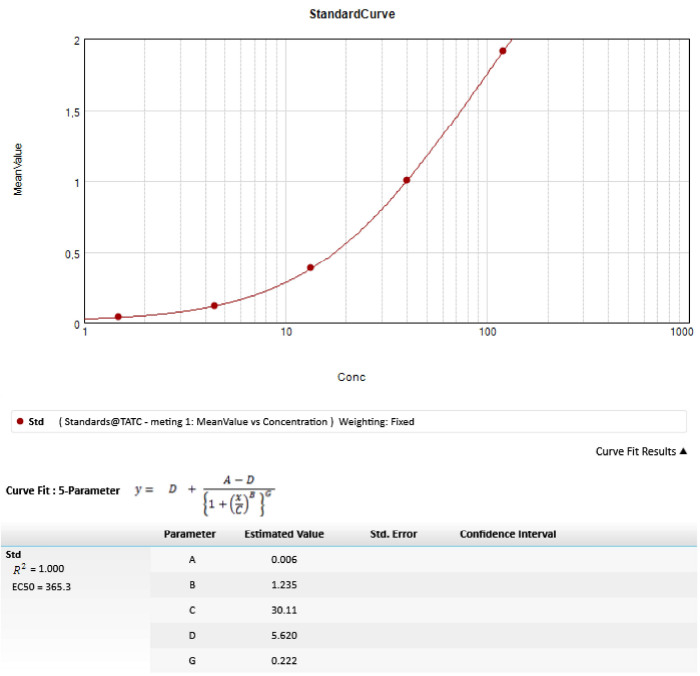
**

**
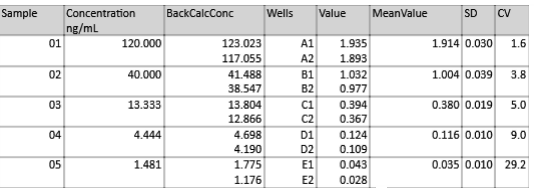
**

**Highest detection limit:** 120 ng/mL

**Lowest detection limit**: 1.5 ng/mL
